# 2D to 3D Modification of Chang–Chang Criterion Considering Multiaxial Coupling Effects in Fiber and Inter-Fiber Directions for Continuous Fiber-Reinforced Composites

**DOI:** 10.3390/polym17172416

**Published:** 2025-09-05

**Authors:** Yingchi Chen, Junhua Guo, Wantao Guo

**Affiliations:** 1 School of Energy and Power, Jiangsu University of Science and Technology, Zhenjiang 212100, China; 2 Luoyang Sunrui Rubber & Plastic Science and Technology Co., Ltd., Luoyang 471000, China; 3 Luoyang Ship Material Research Institute, Luoyang 471000, China

**Keywords:** fiber-reinforced composites, Chang–Chang failure criterion, three-dimensional stress state, failure mode coupling

## Abstract

Fiber-reinforced composites are widely used in aerospace and other fields due to their excellent specific strength, specific stiffness, and corrosion resistance, and further study of their failure criteria is essential to improve the accuracy and reliability of failure behavior prediction under complex loads. There are still some limitations in the current composite failure criterion research, mainly reflected in the lack of promotion of three-dimensional stress state, lack of sufficient consideration of multi-modal coupling effects, and the applicability of the criteria under multiaxial stress and complex loading conditions, which limit the wider application of composites in the leading-edge fields to a certain degree. In this work, a generalized Mohr failure envelope function approach is adopted to obtain the stress on the failure surface as a power series form of independent variable, and the unknown coefficients are determined according to the damage conditions, to extend the Chang–Chang criterion to the three-dimensional stress state, and to consider the coupling effect between the fiber and matrix failure modes. The modified Chang–Chang criterion significantly enhances the failure prediction accuracy of composite materials under complex stress states, especially in the range of multi-axial loading and small off-axis angles, which provides a more reliable theoretical basis and practical guidance for the safe design and performance optimization of composite structures in aerospace and other engineering fields.

## 1. Introduction

Fiber-reinforced composites are formed by physically or chemically combining two or more constituent materials with distinct properties. While retaining the characteristics of each component, this new material exhibits overall performance superior to that of individual materials through complementary synergistic effects, granting it unparalleled advantages in certain applications [1,2,3,4,5,6]. Accurately, predicting the failure behavior of such composites is particularly critical. It not only impacts structural safety, process optimization, and new material development but also serves as a prerequisite for fully leveraging their performance advantages [5,7,8,9]. However, due to the complex interactions between composite components and the multi-mechanism coupled failure processes, establishing universal and precise failure criteria remains a long-standing scientific challenge. This challenge has driven extensive research efforts, most notably the World-Wide Failure Exercises (WWFE-I through III) conducted by Hinton and Kaddour [10,11,12], which established rigorous benchmarks for assessing failure theories and earned recognition as the “Olympics of damage analysis” in composites research [13].

Theories used to describe the failure behavior of fiber-reinforced composites can be divided into two main categories: failure criteria based on image-only science and those based on physical mechanisms. The failure criteria based on image-based theory do not consider the failure modes of the material and usually use mathematical expressions containing polynomial and tensor criteria to describe the failure surface of the material. Typical image-only failure criteria include the Tsai–Hill failure criterion [14], the Hoffman failure criterion [14], the Franklin criterion [15], and the Tsai–Wu tensor failure criterion [16]. The physical mechanism-based criteria, on the other hand, characterize the material’s damage evolution by establishing discriminant equations for different damage modes. Typical physical mechanism-based failure criteria include Chang–Chang criterion [17,18,19], Christensen criterion [20], Puck criterion [21,22], and Nu–Daniel criterion [23,24,25,26].

The Chang–Chang criterion [17,18,19] demonstrates advantages in modeling composite damage under dynamic loading by integrating Hahn–Tsai’s shear nonlinearity [27] and in situ strength concepts. It addresses fiber/matrix failures through progressive damage with property degradation, enabling full failure simulation from initial loading to final failure. However, as engineering applications impose increasingly stringent demands on composite structural design, the limitations of the traditional Chang–Chang criterion in predicting multiaxial failure have become increasingly apparent. This criterion considers stress states solely in-plane, neglecting the coupled effects between transverse and longitudinal failure modes. Under multiaxial loading conditions (e.g., simultaneous tensile stress in both transverse and longitudinal directions), transverse failure induces localized stress concentration, significantly accelerating longitudinal failure progression. This coupled behavior decisively influences the overall damage evolution and ultimate failure mode of the material. Comparative analysis reveals that the original Chang–Chang criterion, failing to account for coupling effects, degenerates into the maximum stress criterion under biaxial stress conditions (resulting in a rectangular failure envelope). In contrast, the improved three-dimensional criterion incorporating coupling effects more accurately fits the experimental data of E-glass/MY750 (yielding an elliptical failure envelope) [28], with particularly pronounced improvements in the stress ranges of $\sigma_{11}>0$ and $\sigma_{22}<0$. This result confirms that the three-dimensional Chang–Chang criterion incorporating coupling effects achieves higher predictive accuracy, demonstrating that multiaxial failure analysis must fully account for the interaction between transverse and longitudinal failure mechanisms.

In recent years, significant breakthroughs have been achieved in both theoretical and applied research on composite material failure criteria, laying the foundation for establishing more robust strength prediction models. Existing research primarily focuses on the following key aspects: Regarding transverse strength prediction, Gu et al. [29] proposed a systematic modeling approach that effectively addresses strength coupling effects under multiaxial stress states through coupled term identification and continuity condition treatment. Li [9] fundamentally revised the unreasonable assumption of “infinite bidirectional compressive strength” in traditional theories, establishing a physically-based transverse strength relationship model. Regarding longitudinal compression failure, Shen et al. [30] innovatively combined Puck-type physical mechanism criteria (particularly the LC-Guo criterion) with multi-level coordinate transformations, significantly enhancing the prediction accuracy of fiber twist failure. Notably, Guo et al.’s [31] optimization method for transverse failure mode characteristic parameters provides crucial reference for establishing cross-scale failure correlations. These research findings reveal two key scientific issues: (1) The failure mechanisms in lateral and longitudinal directions exhibit fundamental differences, necessitating distinct physical modeling approaches. (2) Failure behavior under multiaxial loading demonstrates significant coupling characteristics.

Based on the above recognitions, this study constructs a generalized stress-damage function [32] at the macroscopic scale according to the Chang–Chang criterion [17,18,19], aiming to extend the original Chang–Chang criterion to three-dimensional space. In contrast to conventional methods—such as assuming infinite transverse hydrostatic strength or relying solely on experimental fitting [33,34]—this work adopts the generalized Mohr failure envelope method proposed by Gu to improve predictions under multiaxial stress. The method expands the failure surface stress $\left(\sigma_{ii},\tau_{ij}\right)$ into a power series form, simplifying some of the terms in the expansion based on coordinate invariance and transverse isotropy properties. The generalization from 2D to 3D stress states is then achieved by determining the coefficients of the partial terms against the original Chang–Chang criterion. To further consider the coupling effect of fiber and matrix failure modes, a coupling term $\sigma_{11}\left(\sigma_{22}+\sigma_{33}\right)$ is introduced into the 3D failure function. Then the remaining unknown coefficients are determined according to the actual damage of the material, and the construction of the failure criterion is finally completed. All validated cases in this study employed thermoset matrix continuous fiber composites. The improved criteria demonstrated effectiveness in predicting composite failure through experimental comparison.

The paper is structured as follows: Section 2 details the failure criterion proposed in this work. Section 3 constructs three-dimensional failure functions for resin matrix and yarn/fiber; Section 4 constructs a three-dimensional failure function that takes into account the coupling of transverse and longitudinal failures; Section 5 evaluates the prediction results and discusses them with respect to the improved failure criterion; and Section 6 enumerates some important conclusions and future work prospects.

## 2. An Overview of the Chang–Chang Criterion

The Chang–Chang criterion is currently widely used and has important theoretical and engineering significance in the study of laminates and impact problems. The Chang–Chang criterion takes into account the nonlinear elastic behavior of the matrix, distinguishing between different failure modes such as matrix cracking, fiber–matrix shear failure, and fiber fracture. At the same time, Hahn–Tsai’s shear nonlinear model (a mechanical model characterizing the shear nonlinear principal structure of laminates) is introduced to take into account the shear nonlinear behavior of the material, and the in situ strength is introduced to establish empirical formulas between the transverse tensile in situ strength $Y_{t,\mathrm{is}}$ and the transverse tensile strength $Y_{t}$ and between the shear in situ strength $S_{12,\mathrm{is}}$ and the shear strength $S_{12}$, separately. The current 2D failure criteria are as follows:

1. Fiber tensile failure or fiber–matrix shear failure:
  (1) $${\left(\frac{\sigma_{11}}{X_{t}}\right)}^{2}+\frac{\frac{\tau_{12}^{2}}{2G_{12}}+\frac{3}{4}\alpha \tau_{12}^{4}}{\frac{S_{12,\mathrm{is}}^{2}}{2G_{12}}+\frac{3}{4}\alpha S_{12,\mathrm{is}}^{2}}=1;$$
2. Fiber compression failure:
  (2) $${\left(\frac{\sigma_{11}}{{\bar{X}}_{c}}\right)}^{2}=1;$$
3. Matrix tensile failure:
  (3) $${\left(\frac{\sigma_{22}}{Y_{t,\mathrm{is}}}\right)}^{2}+\frac{\frac{\tau_{12}^{2}}{2G_{12}}+\frac{3}{4}\alpha \tau_{12}^{4}}{\frac{S_{12,\mathrm{is}}^{2}}{2G_{12}}+\frac{3}{4}\alpha S_{12,\mathrm{is}}^{2}}=1;$$
4. Matrix compression failure:

(4) $${\left(\frac{\sigma_{22}}{Y_{c}}\right)}^{2}+\frac{\frac{\tau_{12}^{2}}{2G_{12}}+\frac{3}{4}\alpha \tau_{12}^{4}}{\frac{S_{12,\mathrm{is}}^{2}}{2G_{12}}+\frac{3}{4}\alpha S_{12,\mathrm{is}}^{2}}=1,$$

where $\sigma_{11}$ is the longitudinal positive stress of the material, $X_{t}$ is the longitudinal tensile strength of the material, $\tau_{12}$ is the in-plane shear strength of the material, $G_{12}$ is the shear modulus of the material, α is a nonlinear constant, ${\bar{X}}_{c}$ is the flexural strength of the fibers, $\sigma_{22}$ is the transverse positive stress of the material, and $Y_{c}$ is the transverse compressive strength of the material, $Y_{t,\mathrm{is}}=Y_{t}\left[1+A\frac{\sin\left(\Delta \theta \right)}{N^{B}}\right]$ and $S_{12,\mathrm{is}}=S_{12}\left[1+A\frac{\sin\left(\Delta \theta \right)}{N^{D}}\right]$.

## 3. Construction of 3D Chang–Chang Criterion

Fiber-reinforced composites in practical engineering are usually in a complex three-dimensional stress state, and the two-dimensional criterion cannot fully reflect their real mechanical behavior. The three-dimensional failure criterion can more accurately describe the failure mechanisms of materials under multidirectional stresses, especially for anisotropic materials and multidirectional loading conditions. This not only improves the accuracy of failure prediction, but also provides a more reliable theoretical basis for engineering design and safety assessment under complex stress states. Meanwhile, fiber-reinforced composites usually contain various damage modes, such as fiber pull-out, matrix cracking, etc. Traditional failure criteria are to consider these failure modes separately, while the actual failure is often the result of the coupling of multiple modes. Therefore, applying a two-dimensional failure criterion to predict damage and ignoring the interaction between failure modes may lead to prediction bias. In this work, based on the generalized Mohr failure envelope function acquisition method proposed by Gu, the stress on the failure surface $\left(\sigma_{ii},\tau_{ij}\right)$ is expanded as an independent variable in the form of a power series, and the unknown coefficients are determined according to the damage conditions, the Chang–Chang criterion are extended to the three-dimensional stress state, and the coupling between the fiber and matrix failure modes is considered.

### 3.1. Construction of 3D Failure Criterion for Matrix

**Figure 1:**
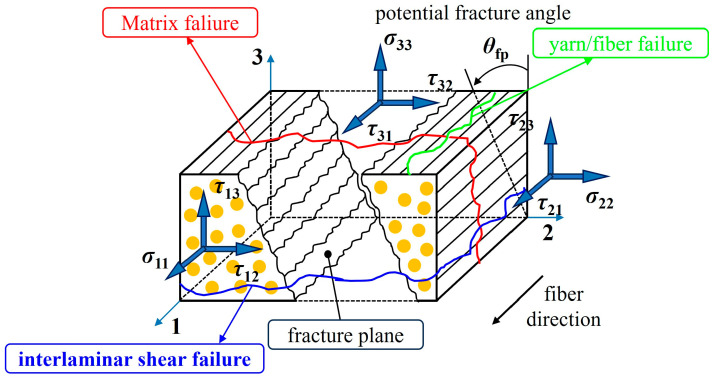
Stress state in the MF plane.

Figure 1 shows the stress distribution in a composite structure and its three main failure modes: fiber failure (labeled in green), matrix failure (labeled in red) and interlaminar failure (labeled in blue). Fiber failure usually occurs in the fiber direction, mainly due to axial stresses $\sigma_{11}$ exceeding the tensile or compressive strength of the fibers; matrix failure arises from shear stresses (e.g., $\tau_{12}$ and $\tau_{13}$) or transverse tensile stresses in the matrix material exceeding its load-bearing capacity; and interlaminar failure is related to the interlaminar shear stresses (e.g., $\tau_{23}$ and $\tau_{21}$), which may lead to delamination phenomena and significantly weaken the overall performance of the structure. In order to better construct the three-dimensional failure function considering the coupling effect of failure modes, this work introduces nine stress components based on Mohr’s failure theory to analyze the failure behavior of composites under different stress states, including the positive stress $\sigma_{11}$ along the fiber direction, the positive stress $\sigma_{22}$ in the transverse direction, and the positive stress $\sigma_{33}$ in the thickness direction, as well as shear stress components in different planes, with $\tau_{12}$, $\tau_{13}$, $\tau_{21}$, $\tau_{23}$, $\tau_{31}$, and $\tau_{32}$.

The Mohr failure envelope function is firstly expanded into the form of a power series with the stress $\left(\sigma_{ii},\tau_{ij}\right)$ on the failure surface as the independent variable:

(5) $$\begin{array}{l} F\left(\sigma_{ii},\tau_{ij}\right)=A_{1}\sigma_{11}+B_{1}\sigma_{22}+C_{1}\sigma_{33}+D_{1}\tau_{12}+E_{1}\tau_{13}+F_{1}\tau_{23}+A_{2}\sigma_{11}^{2}+B_{2}\sigma_{22}^{2}+\\ C_{2}\sigma_{33}^{2}+D_{2}\tau_{12}^{2}+E_{2}\tau_{13}^{2}+F_{2}\tau_{23}^{2}+G_{2}\sigma_{11}\sigma_{22}+H_{2}\sigma_{11}\sigma_{33}+I_{2}\sigma_{11}\tau_{12}+J_{2}\sigma_{11}\tau_{13}+\\ K_{2}\sigma_{11}\tau_{23}+L_{2}\sigma_{22}\sigma_{33}+M_{2}\sigma_{22}\tau_{12}+N_{2}\sigma_{22}\tau_{13}+O_{2}\sigma_{22}\tau_{23}+P_{2}\sigma_{33}\tau_{12}+Q_{2}\sigma_{33}\tau_{13}+\\ R_{2}\sigma_{33}\tau_{23}+S_{2}\tau_{12}\tau_{13}+T_{2}\tau_{12}\tau_{23}+U_{2}\tau_{13}\tau_{23}+ο\left(\sigma_{ii}\right)+ο\left(\tau_{ij}\right)=1 \end{array},$$

where $ο\left(\sigma_{ii}\right)$ and $ο\left(\tau_{ij}\right)$ represent three and more higher-order terms and $A_{1}$, $B_{1}$, $C_{1}$, $D_{1}$, $E_{1}$, $F_{1}$, $A_{2}$, $B_{2}$, $C_{2}$, $D_{2}$, $E_{2}$, $F_{2}$, $G_{2}$, $H_{2}$, $I_{2}$, $J_{2}$, $K_{2}$, $L_{2}$, $M_{2}$, $N_{2}$, $O_{2}$, $P_{2}$, $Q_{2}$, $R_{2}$, $S_{2}$, $T_{2}$, and $U_{2}$ are the coefficients to be determined.

Since the stress $\left(\sigma_{ii},\tau_{ij}\right)$ on the failure surface does not change with rotation of the coordinate system, the failure function (5) satisfies the invariance condition under coordinate transformation. Whether the shear stress $\tau_{ij}$ is positive or negative, its effect on material failure is the same, so the coefficients of the singular sub-linear terms $\tau_{ij}$ and $\tau_{ij}$ in the functional expression must be zero, thus we have the following:

(6) $$\begin{array}{l} \;F\left(\sigma_{ii},\tau_{ij}\right)=A_{1}\sigma_{11}+B_{1}\sigma_{22}+C_{1}\sigma_{33}+A_{2}\sigma_{11}^{2}+B_{2}\sigma_{22}^{2}+C_{2}\sigma_{33}^{2}+D_{2}\tau_{12}^{2}+\\ E_{2}\tau_{13}^{2}+F_{2}\tau_{23}^{2}+G_{2}\sigma_{11}\sigma_{22}+H_{2}\sigma_{11}\sigma_{33}+L_{2}\sigma_{22}\sigma_{33}+ο\left(\sigma_{ii}\right)+ο\left(\tau_{ij}\right)=1 \end{array},$$

In unidirectional laminates, mechanical parameters such as modulus of elasticity, shear modulus, and Poisson’s ratio tend to be the same in the transverse and thickness directions because the distribution of the matrix in these directions is homogeneous and isotropic, and the fibers do not provide significant reinforcement in these two directions. This homogeneous response dominated by the matrix makes unidirectional laminates exhibit isotropic properties in the transverse view, so that there are $B_{1}=C_{1}$, $B_{2}=C_{2}$, and $D_{2}=E_{2}$. Therefore, the failure function in 3D stress state considering coupled longitudinal and transverse failures can be written as follows:

(7) $$\begin{array}{l} F\left(\sigma_{ii},\tau_{ij}\right)=A_{1}\sigma_{11}+B_{1}\left(\sigma_{22}+\sigma_{33}\right)+A_{2}\sigma_{11}^{2}+B_{2}\left(\sigma_{22}^{2}+\sigma_{33}^{2}\right)+D_{2}\left(\tau_{12}^{2}+\tau_{13}^{2}\right)\\ +F_{2}\tau_{23}^{2}+G_{2}\sigma_{11}\left(\sigma_{22}+\sigma_{33}\right)+L_{2}\sigma_{22}\sigma_{33}+ο\left(\sigma_{ii}\right)+ο\left(\tau_{ij}\right)=1 \end{array}.$$

Since the Chang–Chang criterion cuts off at the $\tau_{12}^{4}$ term and there are no three and more than three terms of $\sigma_{ii}$, Equation (7) can be rewritten as follows:

(8) $$\begin{array}{l} F\left(\sigma_{ii},\tau_{ij}\right)=A_{1}\sigma_{11}+B_{1}\left(\sigma_{22}+\sigma_{33}\right)+A_{2}\sigma_{11}^{2}+B_{2}\left(\sigma_{22}^{2}+\sigma_{33}^{2}\right)+\\ D_{2}\left(\tau_{12}^{2}+\tau_{13}^{2}\right)+F_{2}\tau_{23}^{2}+G_{2}\sigma_{11}\left(\sigma_{22}+\sigma_{33}\right)+L_{2}\sigma_{22}\sigma_{33}+ο\left(\tau_{12}^{2}\right)=1 \end{array},$$

where $ο\left(\tau_{12}^{2}\right)=\frac{\left(3\alpha G_{12}\right)}{2S_{12,\mathrm{is}}^{2}+3\alpha G_{12}S_{12,\mathrm{is}}^{4}}\tau_{12}^{4}$. This approach of truncating higher-order terms has been widely adopted in composite material failure modeling. Extensive research and practice demonstrate that [35,36], while ensuring engineering accuracy, reasonable truncation can significantly reduce model complexity without causing significant prediction errors.

Since it is a single matrix failure, all the terms containing $\sigma_{11}$ are omitted, and the 3D matrix failure function is constructed according to Equation (8):

(9) $$F_{m}=A_{1}\left(\sigma_{22}+\sigma_{33}\right)+A_{2}\left(\sigma_{22}^{2}+\sigma_{33}^{2}\right)+B_{2}\left(\tau_{12}^{2}+\tau_{13}^{2}\right)+C_{2}\tau_{23}^{2}+k\cdot \sigma_{22}\sigma_{33}+ο\left(\tau_{12}^{2}\right)=1,$$

where $A_{1}$, $A_{2}$, $B_{2}$, $C_{2}$, and k are coefficients to be determined. $ο\left(\tau_{12}^{2}\right)$ as a higher order infinitesimal can be viewed as 0 when determining the coefficients. The in-plane pure shear test in the 23 direction is given by Equation (9): $C_{2}S_{23}^{2}=1$ can be obtained as $C_{2}=\frac{1}{S_{23}^{2}}$.

Since the matrix usually exhibits brittle fracture in tension and may exhibit shear yielding or plastic deformation in compression, this significant difference in strength, stiffness, and failure modes in tension and compression can more accurately describe the failure behavior of resins under complex stress states, thus improving the prediction accuracy of the failure criteria, and providing a more reliable theoretical basis for the structural design and performance optimization of composites. The matrix compression failure modes in this work are divided into two cases: compression and tensile:

1. Matrix compression:

According to Equation (4) it can be determined that $A_{1}=0$, $A_{2}=\frac{1}{Y_{c}^{2}}$, and $B_{2}=\frac{2}{2S_{12,\mathrm{is}}^{2}+3\alpha \cdot G_{12}\cdot S_{12,\mathrm{is}}^{4}}$. Consider the bidirectional compressive stress state $\sigma_{22}=\sigma_{33}=-p=-Y_{\mathrm{cbi}}$, where $Y_{\mathrm{cbi}}=\left(1-\lambda +\sqrt{\lambda^{2}-\lambda +1}\right)Y_{c}$ and $\lambda =\frac{Y_{t}}{Y_{c}}$ [9], and substitute $A_{1}$, $A_{2}$, $B_{2}$, and $\sigma_{22}=\sigma_{33}=-p=-Y_{\mathrm{cbi}}$ into Equation (9), and we can get $k=\frac{1}{Y_{\mathrm{cbi}}^{2}}-\frac{2}{Y_{c}^{2}}$.

**Figure 2:**
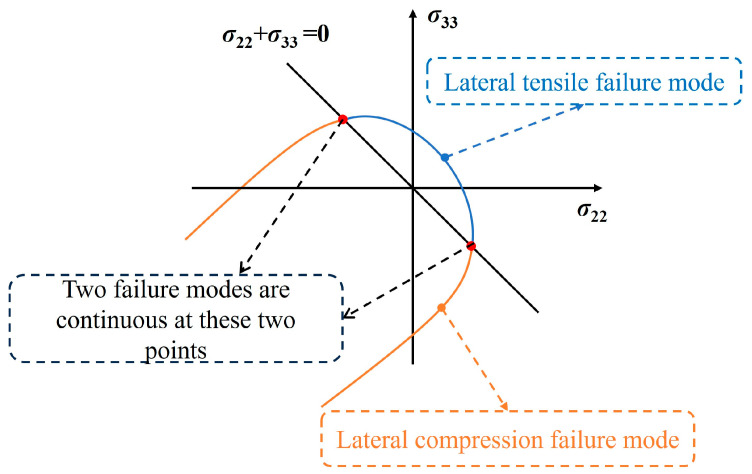
Envelope curve at $\left(\sigma_{22},\;\sigma_{33}\right)$ stress state.

2. Matrix tension:

According to Equation (3) it can be determined that $A_{1}=0$, $A_{2}=\frac{1}{Y_{t,\mathrm{is}}^{2}}$, and $B_{2}=\frac{2}{2S_{12,\mathrm{is}}^{2}+3\alpha \cdot G_{12}\cdot S_{12,\mathrm{is}}^{4}}$; as Figure 2 considers the continuity of the envelope curves for both matrix tensile and matrix compression failure modes in the $\left(\sigma_{22},\;\sigma_{33}\right)$ stress state, when $\sigma_{22}+\sigma_{33}=0$, $F_{\mathrm{mt}}=F_{\mathrm{mc}}$, and $\frac{2\sigma_{22}^{2}}{Y_{c}^{2}}-\left(\frac{1}{Y_{\mathrm{cbi}}^{2}}-\frac{2}{Y_{c}^{2}}\right)\sigma_{22}^{2}=\frac{2\sigma_{22}^{2}}{Y_{t,\mathrm{is}}^{2}}+k\sigma_{22}^{2}$ are obtained, finally it can be obtained that as follows: $k=\frac{1}{Y_{\mathrm{cbi}}^{2}}+\frac{2}{Y_{t,\mathrm{is}}^{2}}-\frac{4}{Y_{c}^{2}}$.

In summary, the 3D failure function for the resin matrix can be written as follows:

(10) $$\left\{\begin{array}{l} F_{\mathrm{mt}}=\frac{\left(\sigma_{22}^{2}+\sigma_{33}^{2}\right)}{Y_{t,\mathrm{is}}^{2}}+\frac{2\left(\tau_{12}^{2}+\tau_{13}^{2}\right)}{\left(2S_{12,\mathrm{is}}^{2}+3\alpha G_{12}S_{12,\mathrm{is}}^{4}\right)}+\frac{\tau_{23}^{2}}{S_{23}^{2}}+\left(\frac{1}{Y_{\mathrm{cbi}}^{2}}+\frac{2}{Y_{t,\mathrm{is}}^{2}}-\frac{4}{Y_{c}^{2}}\right)\sigma_{22}\sigma_{33}+ο\left(\tau_{12}^{2}\right)=1\\ F_{\mathrm{mc}}=\frac{\left(\sigma_{22}^{2}+\sigma_{33}^{2}\right)}{Y_{c}^{2}}+\frac{2\left(\tau_{12}^{2}+\tau_{13}^{2}\right)}{\left(2S_{12,\mathrm{is}}^{2}+3\alpha G_{12}S_{12,\mathrm{is}}^{4}\right)}+\frac{\tau_{23}^{2}}{S_{23}^{2}}+\left(\frac{1}{Y_{\mathrm{cbi}}^{2}}-\frac{2}{Y_{c}^{2}}\right)\sigma_{22}\sigma_{33}+ο\left(\tau_{12}^{2}\right)=1 \end{array}\right.,$$

where $ο\left(\tau_{12}^{2}\right)=\frac{\left(3\alpha G_{12}\right)}{2S_{12,\mathrm{is}}^{2}+3\alpha G_{12}S_{12,\mathrm{is}}^{4}}\tau_{12}^{4}$. The subscript m indicates that the failure function is for the matrix tension, and t and c indicate the two failure behaviors of tensile and compression, respectively.

### 3.2. Construction of 3D Failure Criterion for Fiber

Since the mechanical behavior of unidirectional laminates can usually be described as transverse isotropic, the transformation of the 2D fiber failure function into a 3D failure function only requires the addition of a $\tau_{13}$ term of the corresponding form after the $\tau_{12}$ term according to Equations (1) and (2), and the 3D failure function for a yarn can be written as follows:

(11) $$\left\{\begin{array}{l} F_{\mathrm{ft}}={\left(\frac{\sigma_{11}}{X_{t}}\right)}^{2}+\frac{2\left(\tau_{12}^{2}+\tau_{13}^{2}\right)}{2S_{12,\mathrm{is}}^{2}+3\alpha G_{12}S_{12,\mathrm{is}}^{4}}+\frac{3\alpha G_{12}\left(\tau_{12}^{4}+\tau_{13}^{4}\right)}{2S_{12,\mathrm{is}}^{2}+3\alpha G_{12}S_{12,\mathrm{is}}^{4}}=1\\ F_{\mathrm{fc}}={\left(\frac{\sigma_{11}}{{\bar{X}}_{c}}\right)}^{2}=1 \end{array}\right.,$$

where the subscript f denotes that it is the failure function for the fiber and t and c denote the two failure behaviors of tension and compression, respectively.

## 4. Construction of 3D Failure Criterion Coupling for Transverse and Longitudinal Failure

In order to couple the two failure modes, a coupling term is added in Equation (8) to simulate the coupling of matrix failure and fiber failure, and the three-dimensional failure function coupling the composite fiber and matrix failure modes is constructed as follows:

(12) $$\begin{array}{l} F\left(\sigma_{11},\sigma_{22},\sigma_{33},\tau_{12},\tau_{13},\tau_{23}\right)=A_{1}\sigma_{11}+B_{1}\left(\sigma_{22}+\sigma_{33}\right)+A_{2}\sigma_{11}^{2}+B_{2}\left(\sigma_{22}^{2}+\sigma_{33}^{2}\right)+\\ C_{2}\left(\tau_{12}^{2}+\tau_{13}^{2}\right)+D_{2}\tau_{23}^{2}+E_{2}\sigma_{22}\sigma_{33}+k\sigma_{11}\left(\sigma_{22}+\sigma_{33}\right)+ο\left(\tau_{12}^{2}\right)=1 \end{array},$$

where $A_{1}\sigma_{11}$ and $A_{2}\sigma_{11}^{2}$ represent the contribution of fiber failure modes to composite failure; $B_{1}\left(\sigma_{22}+\sigma_{33}\right)$, $B_{2}\left(\sigma_{22}^{2}+\sigma_{33}^{2}\right)$, $C_{2}\left(\tau_{12}^{2}+\tau_{13}^{2}\right)$, $D_{2}\tau_{23}^{2}$ and $E_{2}\sigma_{22}\sigma_{33}$ represent the contribution of matrix failure modes to composite failure; and $k\sigma_{11}\left(\sigma_{22}+\sigma_{33}\right)$ represents the contribution of mutual coupling between fiber and matrix failure modes to failure.

Unidirectional fiber-reinforced composites have significant anisotropic properties, as shown by the fact that the strength in the fiber direction (longitudinal strength) is usually much higher than the transverse strength (strength perpendicular to the fiber direction). Therefore, the positive stress $\sigma_{11}$ in the fiber direction and the transverse stresses $\sigma_{22}+\sigma_{33}$ have completely different effects on the failure behavior of the material. By comparing the magnitudes of $\sigma_{11}$ and $\sigma_{22}+\sigma_{33}$, it is possible to make a preliminary judgment of the specific failure modes that occur in the transverse and longitudinal directions. After determining the dominant failure mode, by comparing the difference between the strengths of the corresponding failure modes in the transverse and longitudinal directions, we can determine which direction is the first to be damaged and thus construct the corresponding failure function. Therefore, in this work, the determination of the unknown coefficients of the three-dimensional failure function for the coupling of transverse and longitudinal failure modes is discussed in the following eight cases.

### 4.1. Determination of Coefficients Under the Conditions of (σ_11_ < 0, σ_22_ + σ_33_ < 0)

Considering the hydrostatic pressure state $\sigma_{11}=\sigma_{22}=\sigma_{33}=-Y_{\mathrm{cbi}}$, it is assumed in many articles that $Y_{\mathrm{cbi}}\to \infty$, and therefore composites do not fail under hydrostatic pressure, but this is not quite accurate. Based on Li’s paper [9], we believe that, within the physically permissible range of fiber-reinforced composites, the limit of $Y_{\mathrm{cbi}}$ can be determined to be $1.47Y_{c}<Y_{\mathrm{cbi}}<2Y_{c}$. Therefore, by comparing the magnitudes of $X_{c}$ and $Y_{\mathrm{cbi}}$, we can determine whether transverse hydrostatic compression failure or longitudinal compression failure has occurred:

1. When $X_{c}>Y_{\mathrm{cbi}}$, transverse hydrostatic compression failure occurs firstly, so that there is the following:
  (13) $$B_{1}\left(\sigma_{22}+\sigma_{33}\right)+B_{2}\left(\sigma_{22}^{2}+\sigma_{33}^{2}\right)+C_{2}\left(\tau_{12}^{2}+\tau_{13}^{2}\right)+D_{2}\tau_{23}^{2}+E_{2}\sigma_{22}\sigma_{33}=1,$$

Thus, the composite failure function can be simplified as follows:

(14) $$F_{1}\left(\sigma_{11},\sigma_{22},\sigma_{33},\tau_{12},\tau_{13},\tau_{23}\right)=A_{1}\sigma_{11}+A_{2}\sigma_{11}^{2}+k_{1}\sigma_{11}\left(\sigma_{22}+\sigma_{33}\right)+1=1,$$

This gives the following:

(15) $$A_{1}\sigma_{11}+A_{2}\sigma_{11}^{2}+k_{1}\sigma_{11}\left(\sigma_{22}+\sigma_{33}\right)=0,$$

Comparing Equation (15) with Equation (2), $A_{1}=0$ and $A_{2}=\frac{1}{{\bar{X}}_{c}^{2}}$ can be determined, and because of $\sigma_{11}=\sigma_{22}=\sigma_{33}=-Y_{\mathrm{cbi}}$, it can be obtained that as follows:

(16) $$k_{1}=-\frac{1}{2{\bar{X}}_{c}^{2}}.$$

2. When $X_{c}<Y_{\mathrm{cbi}}$, longitudinal compression failure occurs firstly, so that there is the following:
  (17) $$A_{1}\sigma_{11}+A_{2}\sigma_{11}^{2}=1,$$

Thus, the composite failure function can be simplified as follows:

(18) $$\begin{array}{l} F_{2}\left(\sigma_{11},\sigma_{22},\sigma_{33},\tau_{12},\tau_{13},\tau_{23}\right)=B_{1}\left(\sigma_{22}+\sigma_{33}\right)+B_{2}\left(\sigma_{22}^{2}+\sigma_{33}^{2}\right)+\\ C_{2}\left(\tau_{12}^{2}+\tau_{13}^{2}\right)+D_{2}\tau_{23}^{2}+E_{2}\sigma_{22}\sigma_{33}+k_{2}\sigma_{11}\left(\sigma_{22}+\sigma_{33}\right)+1=1 \end{array},$$

This gives the following:

(19) $$B_{1}\left(\sigma_{22}+\sigma_{33}\right)+B_{2}\left(\sigma_{22}^{2}+\sigma_{33}^{2}\right)+C_{2}\left(\tau_{12}^{2}+\tau_{13}^{2}\right)+D_{2}\tau_{23}^{2}+E_{2}\sigma_{22}\sigma_{33}+k_{2}\sigma_{11}\left(\sigma_{22}+\sigma_{33}\right)=0,$$

Comparing Equation (19) with Equation (4), $B_{1}=0$, $B_{2}=\frac{1}{Y_{c}^{2}}$, $C_{2}=\frac{2}{2S_{12,\mathrm{is}}^{2}+3\alpha \cdot G_{12}\cdot S_{12,\mathrm{is}}^{4}}$, $D_{2}=\frac{1}{S_{23}^{2}}$, and $E_{2}=\frac{1}{Y_{\mathrm{cbi}}^{2}}-\frac{2}{Y_{c}^{2}}$ can be determined, and because of $\sigma_{11}=\sigma_{22}=\sigma_{33}=-X_{c}$, it can be obtained that as follows:

(20) $$k_{2}=-\frac{1}{2Y_{\mathrm{cbi}}^{2}}.$$

### 4.2. Determination of Coefficients Under the Conditions of (σ_11_ ≥ 0, σ_22_ + σ_33_ < 0)

Considering the three-way stress state $\left(\sigma_{11}>0,\sigma_{22}=\sigma_{33}<0,\left|\sigma_{11}\right|=\left|\sigma_{22}\right|=\left|\sigma_{33}\right|\right)$, since there is a stress component $\sigma_{22}=\sigma_{33}<0$ acting on the potential matrix failure surface and a stress component $\sigma_{11}>0$ acting on the potential fiber failure surface, it is possible that both transverse compression failure and longitudinal tensile failure may occur in the composite material under this stress state:

1. When $X_{t}>Y_{\mathrm{cbi}}$, transverse hydrostatic compression failure occurs firstly, so that there is the following:
  (21) $$B_{1}\left(\sigma_{22}+\sigma_{33}\right)+B_{2}\left(\sigma_{22}^{2}+\sigma_{33}^{2}\right)+C_{2}\left(\tau_{12}^{2}+\tau_{13}^{2}\right)+D_{2}\tau_{23}^{2}+E_{2}\sigma_{22}\sigma_{33}=1,$$

Thus, the composite failure function can be simplified as follows:

(22) $$F_{3}\left(\sigma_{11},\sigma_{22},\sigma_{33},\tau_{12},\tau_{13},\tau_{23}\right)=A_{1}\sigma_{11}+A_{2}\sigma_{11}^{2}+k_{3}\sigma_{11}\left(\sigma_{22}+\sigma_{33}\right)+1=1,$$

This gives the following:

(23) $$A_{1}\sigma_{11}+A_{2}\sigma_{11}^{2}+k_{3}\sigma_{11}\left(\sigma_{22}+\sigma_{33}\right)=0.$$

Comparing Equation (23) with Equation (1), $A_{1}=0$ and $A_{2}=\frac{1}{X_{t}^{2}}$ can be determined, and because of $\sigma_{11}=Y_{\mathrm{cbi}}$ and $\sigma_{22}=\sigma_{33}=-Y_{\mathrm{cbi}}$, it can be obtained that as follows:

(24) $$k_{3}=\frac{1}{2X_{t}^{2}}.$$

2. When $X_{t}<Y_{\mathrm{cbi}}$, longitudinal tensile failure occurs preferentially, so that there is the following:
  (25) $$A_{1}\sigma_{11}+A_{2}\sigma_{11}^{2}=1,$$

Thus, the composite failure function can be simplified as follows:

(26) $$\begin{array}{l} F_{4}\left(\sigma_{11},\sigma_{22},\sigma_{33},\tau_{12},\tau_{13},\tau_{23}\right)=B_{1}\left(\sigma_{22}+\sigma_{33}\right)+B_{2}\left(\sigma_{22}^{2}+\sigma_{33}^{2}\right)+\\ C_{2}\left(\tau_{12}^{2}+\tau_{13}^{2}\right)+D_{2}\tau_{23}^{2}+E_{2}\sigma_{22}\sigma_{33}+k_{4}\sigma_{11}\left(\sigma_{22}+\sigma_{33}\right)+1=1 \end{array},$$

This gives the following:

(27) $$B_{1}\left(\sigma_{22}+\sigma_{33}\right)+B_{2}\left(\sigma_{22}^{2}+\sigma_{33}^{2}\right)+C_{2}\left(\tau_{12}^{2}+\tau_{13}^{2}\right)+D_{2}\tau_{23}^{2}+E_{2}\sigma_{22}\sigma_{33}+k_{4}\sigma_{11}\left(\sigma_{22}+\sigma_{33}\right)=0,$$

Comparing Equation (27) with Equation (4), $B_{1}=0$, $B_{2}=\frac{1}{Y_{c}^{2}}$, $C_{2}=\frac{2}{2S_{12,\mathrm{is}}^{2}+3\alpha \cdot G_{12}\cdot S_{12,\mathrm{is}}^{4}}$, $D_{2}=\frac{1}{S_{23}^{2}}$, $E_{2}=\frac{1}{Y_{\mathrm{cbi}}^{2}}-\frac{2}{Y_{c}^{2}}$, $\sigma_{11}=X_{t}$, and $\sigma_{22}=\sigma_{33}=-X_{t}$ can be determined, and thus it can be obtained that as follows:

(28) $$k_{4}=\frac{1}{2Y_{\mathrm{cbi}}^{2}}.$$

### 4.3. Determination of Coefficients Under the Conditions of (σ_11_ < 0, σ_22_ + σ_33_ ≥ 0)

The following explains how to determine coefficients under the conditions of (σ_11_ < 0, σ_22_ + σ_33_ ≥ 0):

1. When $X_{c}>Y_{t}$, transverse tensile failure occurs preferentially, so that there is the following:
  (29) $$B_{1}\left(\sigma_{22}+\sigma_{33}\right)+B_{2}\left(\sigma_{22}^{2}+\sigma_{33}^{2}\right)+C_{2}\left(\tau_{12}^{2}+\tau_{13}^{2}\right)+D_{2}\tau_{23}^{2}+E_{2}\sigma_{22}\sigma_{33}=1,$$

Thus, the composite failure function can be simplified as follows:

(30) $$F_{5}\left(\sigma_{11},\sigma_{22},\sigma_{33},\tau_{12},\tau_{13},\tau_{23}\right)=A_{1}\sigma_{11}+A_{2}\sigma_{11}^{2}+k_{5}\sigma_{11}\left(\sigma_{22}+\sigma_{33}\right)+1=1,$$

This gives the following:

(31) $$A_{1}\sigma_{11}+A_{2}\sigma_{11}^{2}+k_{5}\sigma_{11}\left(\sigma_{22}+\sigma_{33}\right)=0,$$

Comparing Equation (31) with Equation (2), $A_{1}=0$, $A_{2}=\frac{1}{{\bar{X}}_{c}^{2}}$, and $\sigma_{11}=-Y_{\mathrm{tbi}}$ can be determined, and because of $\sigma_{22}=\sigma_{33}=Y_{\mathrm{tbi}}$, it can be obtained that as follows:

(32) $$k_{5}=\frac{1}{2{\bar{X}}_{c}^{2}}.$$

2. When $X_{c}<Y_{t}$, longitudinal compression failure occurs preferentially, so that there is the following:
  (33) $$A_{1}\sigma_{11}+A_{2}\sigma_{11}^{2}=1,$$

Thus, the composite failure function can be simplified as follows:

(34) $$\begin{array}{l} F_{6}\left(\sigma_{11},\sigma_{22},\sigma_{33},\tau_{12},\tau_{13},\tau_{23}\right)=B_{1}\left(\sigma_{22}+\sigma_{33}\right)+B_{2}\left(\sigma_{22}^{2}+\sigma_{33}^{2}\right)+\\ C_{2}\left(\tau_{12}^{2}+\tau_{13}^{2}\right)+D_{2}\tau_{23}^{2}+E_{2}\sigma_{22}\sigma_{33}+k_{6}\sigma_{11}\left(\sigma_{22}+\sigma_{33}\right)+1=1 \end{array},$$

This gives the following:

(35) $$B_{1}\left(\sigma_{22}+\sigma_{33}\right)+B_{2}\left(\sigma_{22}^{2}+\sigma_{33}^{2}\right)+C_{2}\left(\tau_{12}^{2}+\tau_{13}^{2}\right)+D_{2}\tau_{23}^{2}+E_{2}\sigma_{22}\sigma_{33}+k_{6}\sigma_{11}\left(\sigma_{22}+\sigma_{33}\right)=0,$$

Comparing Equation (35) with Equation (3), $B_{1}=0$, $B_{2}=\frac{1}{Y_{t,\mathrm{is}}^{2}}$, $C_{2}=\frac{2}{2S_{12,\mathrm{is}}^{2}+3\alpha \cdot G_{12}\cdot S_{12,\mathrm{is}}^{4}}$, $D_{2}=\frac{1}{S_{23}^{2}}$, $E_{2}=\frac{1}{Y_{\mathrm{cbi}}^{2}}+\frac{2}{Y_{t,\mathrm{is}}^{2}}-\frac{4}{Y_{c}^{2}}$, $\sigma_{11}=-X_{c}$, and $\sigma_{22}=\sigma_{33}=X_{c}$ can be determined, and thus it can be obtained that as follows:

(36) $$k_{6}=\frac{1}{2Y_{\mathrm{cbi}}^{2}}+\frac{2}{Y_{t,\mathrm{is}}^{2}}-\frac{2}{Y_{c}^{2}}.$$

### 4.4. Determination of Coefficients Under the Conditions of (σ_11_ ≥ 0, σ_22_ + σ_33_ ≥ 0)

The following explains how to determine the coefficients under the conditions of (σ_11_ ≥ 0, σ_22_ + σ_33_ ≥ 0):

1. When $X_{t}>Y_{t}$, transverse stretch failure occurs preferentially, so that there is the following:
  (37) $$B_{1}\left(\sigma_{22}+\sigma_{33}\right)+B_{2}\left(\sigma_{22}^{2}+\sigma_{33}^{2}\right)+C_{2}\left(\tau_{12}^{2}+\tau_{13}^{2}\right)+D_{2}\tau_{23}^{2}+E_{2}\sigma_{22}\sigma_{33}=1,$$

Thus, the composite failure function can be simplified as follows:

(38) $$F_{7}\left(\sigma_{11},\sigma_{22},\sigma_{33},\tau_{12},\tau_{13},\tau_{23}\right)=A_{1}\sigma_{11}+A_{2}\sigma_{11}^{2}+k_{7}\sigma_{11}\left(\sigma_{22}+\sigma_{33}\right)+1=1,$$

This gives the following:

(39) $$A_{1}\sigma_{11}+A_{2}\sigma_{11}^{2}+k_{7}\sigma_{11}\left(\sigma_{22}+\sigma_{33}\right)=0,$$

Comparing Equation (39) with Equation (1), $A_{1}=0$ and $A_{2}=\frac{1}{X_{t}^{2}}$ can be determined, and because of $\sigma_{11}=\sigma_{22}=\sigma_{33}=Y_{\mathrm{tbi}}$, it can be obtained that as follows:

(40) $$k_{7}=-\frac{1}{2X_{t}^{2}}.$$

2. When $X_{t}<Y_{t}$, longitudinal tensile failure occurs preferentially, so that there is the following:
  (41) $$A_{1}\sigma_{11}+A_{2}\sigma_{11}^{2}=1,$$

Thus, the composite failure function can be simplified as follows:

(42) $$\begin{array}{l} F_{8}\left(\sigma_{11},\sigma_{22},\sigma_{33},\tau_{12},\tau_{13},\tau_{23}\right)=B_{1}\left(\sigma_{22}+\sigma_{33}\right)+B_{2}\left(\sigma_{22}^{2}+\sigma_{33}^{2}\right)+\\ C_{2}\left(\tau_{12}^{2}+\tau_{13}^{2}\right)+D_{2}\tau_{23}^{2}+E_{2}\sigma_{22}\sigma_{33}+k_{8}\sigma_{11}\left(\sigma_{22}+\sigma_{33}\right)+1=1 \end{array},$$

This gives the following:

(43) $$B_{1}\left(\sigma_{22}+\sigma_{33}\right)+B_{2}\left(\sigma_{22}^{2}+\sigma_{33}^{2}\right)+C_{2}\left(\tau_{12}^{2}+\tau_{13}^{2}\right)+D_{2}\tau_{23}^{2}+E_{2}\sigma_{22}\sigma_{33}+k_{8}\sigma_{11}\left(\sigma_{22}+\sigma_{33}\right)=0,$$

Comparing Equation (43) with Equation (3), $B_{1}=0$, $B_{2}=\frac{1}{Y_{t,\mathrm{is}}^{2}}$, $C_{2}=\frac{2}{2S_{12,\mathrm{is}}^{2}+3\alpha \cdot G_{12}\cdot S_{12,\mathrm{is}}^{4}}$, $D_{2}=\frac{1}{S_{23}^{2}}$, $E_{2}=\frac{1}{Y_{\mathrm{cbi}}^{2}}+\frac{2}{Y_{t,\mathrm{is}}^{2}}-\frac{4}{Y_{c}^{2}}$, and $\sigma_{11}=\sigma_{22}=\sigma_{33}=X_{t}$ can be determined, and thus it can be obtained that as follows:

(44) $$k_{8}=-\frac{1}{2Y_{\mathrm{cbi}}^{2}}-\frac{2}{Y_{t,\mathrm{is}}^{2}}+\frac{2}{Y_{c}^{2}}.$$

### 4.5. Theoretical Justification of Stress-State-Coupled Transition at Failure Boundaries

The abrupt change in coupling coefficients exhibited by composite materials during failure stems from micro-scale interaction mechanisms between fiber and matrix failure modes. Although, at the macro-scale, material failure criteria are only triggered when cumulative fiber fractures reach a critical threshold, at the micro-scale, early fractures in some fibers begin to influence the material’s overall mechanical behavior. This micro-damage reduces the material’s resistance to matrix failure, while the resulting localized matrix damage may propagate along fiber directions, further diminishing the material’s capacity to resist fiber failure. This complex bidirectional coupling naturally leads to significant variations in material coupling behavior under different stress states, constituting the physical foundation for the coupling coefficient discontinuity observed in our theoretical modeling. By accounting for this mesoscopic damage evolution process, the abrupt behavior exhibited by materials at stress state boundaries can be reasonably explained from a physical perspective.

### 4.6. Summary of the Modified Chang–Chang Criterion

Following the preceding derivations, all coefficients have been determined. The modified Chang–Chang criterion is now presented by distinguishing different stress states. Furthermore, it should be specifically noted that the transverse hydrostatic tensile strength and transverse hydrostatic compressive strength of composite materials are difficult to obtain in practice. Therefore, in the subsequent summary of criterion expressions, a function form taking the minimum value is adopted. Subscripts ∥ and ⊥ represent longitudinal compressive failure and transverse compressive failure, respectively, while superscripts C and T denote compression and tension, respectively:

1. In the case of σ_11_ < 0 and σ_22_ + σ_33_ < 0
  (45) $$\left\{\begin{array}{l} F_{∥⊥}^{\mathrm{CC}}=F_{∥}^{C}+F_{⊥}^{C}+\min\left\{-\frac{1}{2{\bar{X}}_{c}^{2}},-\frac{1}{2Y_{\mathrm{cbi}}^{2}}\right\}\cdot \sigma_{11}\left(\sigma_{22}+\sigma_{33}\right)=1\\ F_{∥}^{C}=\frac{\sigma_{11}^{2}}{{\bar{X}}_{c}^{2}}\\ F_{⊥}^{C}=\frac{\left(\sigma_{22}^{2}+\sigma_{33}^{2}\right)}{Y_{c}^{2}}+\frac{2\left(\tau_{12}^{2}+\tau_{13}^{2}\right)}{\left(2S_{12,\mathrm{is}}^{2}+3\alpha G_{12}S_{12,\mathrm{is}}^{4}\right)}+\frac{\tau_{23}^{2}}{S_{23}^{2}}+\left(\frac{1}{Y_{\mathrm{cbi}}^{2}}-\frac{2}{Y_{c}^{2}}\right)\cdot \sigma_{22}\sigma_{33}+ο\left(\tau_{12}^{2}\right) \end{array}\right..$$

If $F_{∥⊥}^{\mathrm{CC}}\geq 1$ and $F_{∥}^{C}>F_{⊥}^{C}$, the composites undergo longitudinal compressive failure; if $F_{∥⊥}^{\mathrm{CC}}\geq 1$ and $F_{∥}^{C}<F_{⊥}^{C}$, the composites undergo transverse compressive failure.

2. In the case of σ_11_ ≥ 0 and σ_22_ + σ_33_ < 0
  (46) $$\left\{\begin{array}{l} F_{∥⊥}^{\mathrm{TC}}=F_{∥}^{T}+F_{⊥}^{c}+\min\left\{\frac{1}{2X_{t}^{2}},\frac{1}{2Y_{\mathrm{cbi}}^{2}}\right\}\cdot \sigma_{11}\left(\sigma_{22}+\sigma_{33}\right)=1\\ F_{∥}^{T}=\frac{\sigma_{11}^{2}}{X_{t}^{2}}\\ F_{⊥}^{c}=\frac{\left(\sigma_{22}^{2}+\sigma_{33}^{2}\right)}{Y_{c}^{2}}+\frac{2\left(\tau_{12}^{2}+\tau_{13}^{2}\right)}{\left(2S_{12,\mathrm{is}}^{2}+3\alpha G_{12}S_{12,\mathrm{is}}^{4}\right)}+\frac{\tau_{23}^{2}}{S_{23}^{2}}+\left(\frac{1}{Y_{\mathrm{cbi}}^{2}}-\frac{2}{Y_{c}^{2}}\right)\cdot \sigma_{22}\sigma_{33}+ο\left(\tau_{12}^{2}\right) \end{array}\right..$$

If $F_{∥⊥}^{\mathrm{TC}}\geq 1$ and $F_{∥}^{T}>F_{⊥}^{c}$, the composites undergo longitudinal compressive failure; if $F_{∥⊥}^{\mathrm{TC}}\geq 1$ and $F_{∥}^{T}<F_{⊥}^{c}$, the composites undergo transverse compressive failure.

3. In the case of σ_11_ ≥ 0 and σ_22_ + σ_33_ ≥ 0
  (47) $$\left\{\begin{array}{l} F_{∥⊥}^{\mathrm{CT}}=F_{∥}^{C}+F_{⊥}^{T}+\min\left\{\frac{1}{2{\bar{X}}_{c}^{2}},\frac{1}{2Y_{\mathrm{cbi}}^{2}}+\frac{2}{Y_{t,\mathrm{is}}^{2}}-\frac{2}{Y_{c}^{2}}\right\}\cdot \sigma_{11}\left(\sigma_{22}+\sigma_{33}\right)=1\\ F_{∥}^{C}=\frac{\sigma_{11}^{2}}{{\bar{X}}_{c}^{2}}\\ F_{⊥}^{T}=\frac{\left(\sigma_{22}^{2}+\sigma_{33}^{2}\right)}{Y_{t,\mathrm{is}}^{2}}+\frac{2\left(\tau_{12}^{2}+\tau_{13}^{2}\right)}{\left(2S_{12,\mathrm{is}}^{2}+3\alpha G_{12}S_{12,\mathrm{is}}^{4}\right)}+\frac{\tau_{23}^{2}}{S_{23}^{2}}+\left(\frac{1}{Y_{\mathrm{cbi}}^{2}}+\frac{2}{Y_{t,\mathrm{is}}^{2}}-\frac{4}{Y_{c}^{2}}\right)\cdot \sigma_{22}\sigma_{33}+ο\left(\tau_{12}^{2}\right) \end{array}\right..$$

If $F_{∥⊥}^{\mathrm{CT}}\geq 1$ and $F_{∥}^{C}>F_{⊥}^{T}$, the composites undergo longitudinal compressive failure; if $F_{∥⊥}^{\mathrm{CT}}\geq 1$ and $F_{∥}^{C}<F_{⊥}^{T}$, the composites undergo transverse compressive failure.

4. In the case of σ_11_ ≥ 0 and σ_22_ + σ_33_ ≥ 0
  (48) $$\left\{\begin{array}{l} F_{∥⊥}^{\mathrm{TT}}=F_{∥}^{T}+F_{⊥}^{T}+\min\left\{-\frac{1}{2X_{t}^{2}},-\frac{1}{2Y_{\mathrm{cbi}}^{2}}-\frac{2}{Y_{t,\mathrm{is}}^{2}}+\frac{2}{Y_{c}^{2}}\right\}\cdot \sigma_{11}\left(\sigma_{22}+\sigma_{33}\right)\\ F_{∥}^{T}=\frac{\sigma_{11}^{2}}{X_{t}^{2}}\\ F_{⊥}^{T}=\frac{\left(\sigma_{22}^{2}+\sigma_{33}^{2}\right)}{Y_{t,\mathrm{is}}^{2}}+\frac{2\left(\tau_{12}^{2}+\tau_{13}^{2}\right)}{\left(2S_{12,\mathrm{is}}^{2}+3\alpha G_{12}S_{12,\mathrm{is}}^{4}\right)}+\frac{\tau_{23}^{2}}{S_{23}^{2}}+\left(\frac{1}{Y_{\mathrm{cbi}}^{2}}+\frac{2}{Y_{t,\mathrm{is}}^{2}}-\frac{4}{Y_{c}^{2}}\right)\cdot \sigma_{22}\sigma_{33}+ο\left(\tau_{12}^{2}\right) \end{array}\right..$$

If $F_{∥⊥}^{\mathrm{TT}}\geq 1$ and $F_{∥}^{T}>F_{⊥}^{T}$, the composites undergo longitudinal compressive failure; if $F_{∥⊥}^{\mathrm{TT}}\geq 1$ and $F_{∥}^{T}<F_{⊥}^{T}$, the composites undergo transverse compressive failure.

## 5. Results and Discussions

Next, experimental data from several composite materials in Table 1 will be selected to evaluate the improved Chang–Chang criterion (see Equation (45)) and compare it with several classical failure criteria. It should be noted that, although the selected materials may not fully represent all fiber–matrix systems, the vast majority of them originate from the internationally recognized World Wide Failure Envelope II (WWFE-II) benchmark database, providing an effective basis for comparison and validation in this study.

### 5.1. Experimental Evaluation of the Modified Chang–Chang Criterion

#### 5.1.1. Performance Under Biaxial Stress States

**Figure 3:**
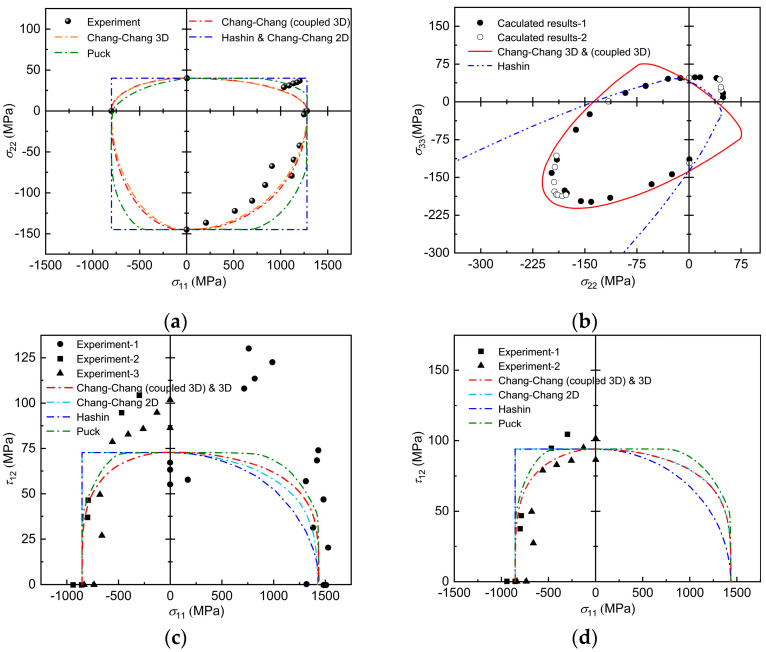
Comparison of predicted failure envelope with experimental results for (**a**) E-glass/MY750 unidirectional plate σ_11_-σ_22_ failure envelope; (**b**) E-glass/LY556 unidirectional plate σ_22_-σ_33_ failure envelope; (**c**) T300/BSL914C unidirectional plate σ_11_-τ_12_ S_12_ = 73 MPa. (**d**) After modification S_12_ = 93.8 MPa.

The first example is the damage envelope of an E-glass/MY750 unidirectional sheet subjected to composite stresses along the matrix direction and along the fiber direction. In the biaxial $\sigma_{11}-\sigma_{22}$ stress state, the Hashin criterion and the original Chang–Chang criterion degenerate into the classical maximum stress criterion. As shown in Figure 3a, the damage envelopes of Hashin and the original Chang–Chang are rectangular in shape, and the values of the upper and lower boundaries correspond to the basic tensile and compressive strengths in the transverse direction, and the values of the left and right boundaries correspond to the basic tensile and compressive strengths in the longitudinal direction, respectively. The Puck failure criterion, the Chang–Chang criterion extended to three-dimensional stress states, and finally the three-dimensional Chang–Chang criterion, which takes into account the interaction of transverse and longitudinal failures, are more accurate in predicting the results for experimental data due to the interaction of fibers. However, the Puck failure criterion overestimated the load carrying capacity of the unidirectional lamellae in the regions $\sigma_{11}\geq 0$ and $\sigma_{22}\geq 0$, leading to a degradation of the Puck failure criterion into a maximum stress criterion component between these two regions as well.

The second example is the damage envelope of an E-glass/LY556 unidirectional sheet subjected to a biaxial transverse composite stress. Based on the numerical data provided in the article by Li [9], and using the recent work by Camanho et al. [39], the damage envelope under biaxial transverse loading is compared with the fine mechanics results as shown in Figure 3b. The proposed criterion obtains more satisfactory predictive power in the overall $\sigma_{22}-\sigma_{33}$ stress space. For the proposed criterion, the error between the numerical data and the damage envelope formed by the conventional uniaxial strength is still acceptable, despite the better agreement with the biaxial strength as an input parameter. Furthermore, both the improved Chang–Chang criterion for failure and Hashin matrix tensile damage criterion overestimate the interaction of the biaxial stresses $\sigma_{22}$ and $\sigma_{33}$, especially in the first quadrant of Figure 3. The most significant difference occurs when a biaxial transverse compression load is applied. The damage trajectory of the Hashin failure criterion is open-ended, which is a natural consequence of the assumption of infinite, isobiaxial, transverse compressive strength (i.e., $Y_{\mathrm{cbi}}\to \infty$) inherent in equation. This empiricism may conflict with computational micromechanics. Instead, the improved Chang–Chang criterion uses the finite intensity of $Y_{\mathrm{cbi}}$, and thus it is a closed curve shaped like an ellipse.

The third example is the failure envelope of the T300/BSL914C unidirectional plate under the $\sigma_{11}-\tau_{12}$ stress state, as shown in Figure 3c,d. The predictions of each criterion deviate considerably from the experimental results, e.g., all the criteria do not predict the data well for the red circles all out. However, if the experimental data are dispersed too much, thus dropping the experimental data that are far away from each criterion and calculating the failure envelope at 93.8 MPa shear strength, the prediction can be improved, as shown in Figure 3d. In this loading case, the damage envelope predicted by the three-dimensional Chang–Chang criterion, which takes into account the interaction of transverse and longitudinal failures, seems to be in better agreement with the experimental data. However, sufficiently accurate experimental data are needed to further validate the theoretical predictions. And the original 2D Chang–Chang criterion still behave as a maximum stress criterion in the region of $\sigma_{11}\leq 0$, so its failure envelope is rectangular. It should be noted that the shear strength parameter S12 was adjusted from 73 MPa to 93.8 MPa to account for the in situ strengthening effect [42,43,44,45]—where constrained layers exhibit enhanced mechanical properties due to crack growth inhibition and stress redistribution mechanisms. This approach will significantly improve the accuracy of failure behavior prediction.

#### 5.1.2. Performance Under Triaxial Stress States

**Figure 4:**
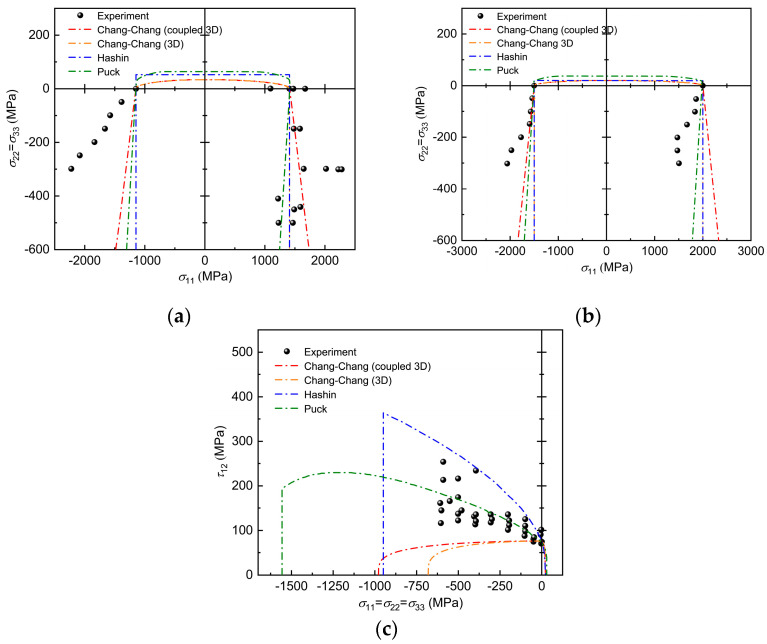
Comparison of predicted failure envelope with experimental results for (**a**) S-glass/epoxy unidirectional plate σ_11_–σ_22_ (σ_22_ = σ_33_) failure envelope; (**b**) A-S carbon/epoxy unidirectional plate σ_11_–σ_22_ (σ_22_ = σ_33_) failure envelope; (**c**) T300/PR319 unidirectional plate (σ_11_ = σ_22_ = σ_33_)–τ_12_ failure envelope.

The failure envelope generated by the failure criteria and the corresponding experimental data are given in Figure 4a,b. The results of the experimental data for this stress state are extracted from the literature [40]. In the presence of transverse pressure, longitudinal tensile and compressive loads are applied to the S-glass/epoxy and A-S carbon/epoxy layers. The failure envelope is not a closed line when $\sigma_{22}=\sigma_{33}$ is negative, and its branches continue until $\sigma_{22}=\sigma_{33}=-\infty$. This phenomenon is due to the fact that, in the absence of other shear stresses, the compressive positive stress $\sigma_{n}$ does not lead to matrix fracture. Especially in the region characterized by triaxial compression and transverse compression and longitudinal stretching, the three-dimensional Chang–Chang criterion considering transverse and longitudinal interactions appeared to have a better predictive ability for the experimental data than the Hashin failure criterion, the Puck failure criterion, and the three-dimensional Chang–Chang criterion, as shown in Figure 4a. However, it can also be seen from Figure 4b that the A-S carbon/epoxy layer performs poorly for all the failure criteria except for the three-dimensional Chang–Chang criterion that considers transverse and longitudinal influences in the case of triaxial compression, which is more accurately predicted. There seems to be a particular point in the red circle in Figure 4b that divides the envelope into two intervals when the experimental error is discarded. Beyond this demarcation point, the interaction between matrix and fiber fracture suddenly becomes apparent. Further studies may need to consider more accurately the damage behavior of the material under hydrostatic pressure. At $\sigma_{22}=\sigma_{33}>0$, the failure envelope of the 3D Chang–Chang criterion, which considers the interaction of transverse and longitudinal failures, matches perfectly with that of the 3D Chang–Chang criterion, whereas the Puck criterion predicts a failure envelope that is not perpendicular due to the additional consideration of Poisson’s effect on transverse stresses. Figure 4c shows the results predicted by each failure criterion for T300/PR319 material plywood under combined hydrostatic and shear stresses compared to the experimental results [41]. All theories of damage predict MF fracture due to the combination of tensile positive and tangential stresses on the fracture surface. In this example, we do not consider the hydrostatic pressure to be tending to infinity, so the improved Chang–Chang criterion is closed curves like the others. Additionally, under the specific stress conditions shown ($\sigma_{11}=\sigma_{22}=\sigma_{33}<0$ and $\tau_{12}>0$), variations in $Y_{\mathrm{cbi}}$ significantly change the shape and range of the envelope curve, playing a vital role in determining the failure envelope. In contrast, both Puck and Hashin’s criteria suggest that as the compression $\sigma_{11}$ increases, the state of applied stress will ultimately lead to the failure of FF. Due to the three-dimensional Chang-Chang criterion and the three-dimensional Chang-Chang criterion considering transverse and longitudinal coupling effects lacks a strengthening effect. Therefore, it cannot exhibit an upward trend during the initial stage of increased compressive stress $\sigma_{11}$, but instead declines directly until failure.

**Figure 5:**
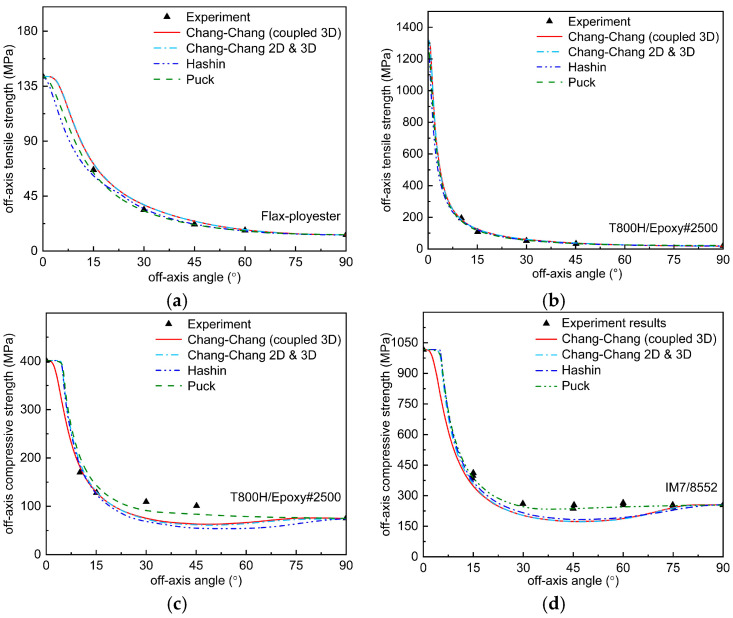
Comparison of predicted failure envelopes with experimental results for (**a**) off-axis tension flax-ployester; (**b**) off-axis tension T800H/Epoxy#2500; (**c**) off-axis compression T800H/Epoxy#2500; (**d**) off-axis compression IM7/8552.

Figure 5a,b show the predicted results of off-axis compressive strength of Flax-ployester and T800H/Epoxy#2500 for different criteria. The stresses on the material when the unidirectional sheet is subjected to off-axis tension are $\sigma_{11}=\sigma \sin\theta^{2}$, $\sigma_{22}=\sigma \cos\theta^{2}$, and $\tau_{12}=\sigma \sin\theta \cdot \cos\theta$. The basic parameters of Flax-ployester and T800H/Epoxy#2500 are shown in Table 1, and the curves of the off-axis tensile damage stresses with off-axis angle θ calculated from each failure criterion are given in Figure 5 and compared with the experimental data. As can be seen from the Figure 5a,b, all the failure criteria, including the results predicted by the Chang–Chang criterion before and after the improvement, fit the experimental data well. Since $\sigma_{33}=0$, the Chang–Chang criterion extended to three dimensions are indistinguishable from the initial two-dimensional criterion. It is only when the off-axis angle exceeds approximately 10° that the failure mode predicted by Hashin, Puck, and the original two-dimensional Chang–Chang criterion shifts from fiber failure to matrix failure. However, experimental studies have found that the failure mode shifts within 5° of off-axis stretching. It is clear that the predictions of the three-dimensional Chang–Chang criterion, which take into account the transverse and longitudinal damage interactions, correlate better with the experimental observations. To quantitatively evaluate the fitting accuracy of each criterion for experimental data, we calculated the prediction error using the following formula: Error = (Predicted load amplitude − Experimental load amplitude)/Experimental load amplitude × 100%. Based on this metric, Table 2 compares the average prediction errors for off-axis tensile strength across different criteria. Table 3 compares the average prediction errors for off-axis compressive strength across different criteria. As can be seen from Table 3, for Flax-ployester and T800H/Epoxy#2500, the prediction error of Puck is the smallest; for T800H/Epoxy#2500, the error predicted by the improved Chang–Chang criterion is not much different from that predicted by Hashin criterion and is significantly better than that predicted by the original Chang–Chang criterion; for Flax-ployester, the difference between the prediction errors of the original and the improved Chang–Chang criterion and the other two criteria is larger, but the prediction accuracy of the three-dimensional Chang–Chang criterion, which takes into account the transverse-longitudinal coupling, is significantly improved compared to the original Chang–Chang criterion.

Figure 5c,d show the predicted results of the off-axis compressive strength of T800H/Epoxy#2500 and IM7/8552 for different criteria. It is clear that not all criteria are capable of predicting the off-axis compressive strength of composites within an engineering acceptable margin of error. From the Figure, it can be seen that only the Puck criterion is better for predicting the experimental data points, and the Hashin criterion and the Chang–Chang criterion before and after the improvement do not give satisfactory predictions. Overall, however, the off-axis compressive strength of the composites predicted by the improved Chang–Chang criterion at off-axis angles less than 30° is in better agreement with the experimental data. In addition, the off-axis compressive strength curves predicted by all the criteria except Puck’s criterion show a tendency to decrease and then increase or even decrease again, while the off-axis tensile strength curves are decreasing. Notably, the improved criterion does not exhibit uniformly superior accuracy across all loading scenarios compared to classical criteria. Its key strength lies in enhancing prediction reliability under multiaxial coupled stress conditions, as demonstrated in other sections of this work, rather than providing unconditional improvements in simpler loading cases such as pure off-axis tension or compression alone—this aligns with the original intent of our modifications. The reason for this phenomenon is that the crack angle changes as the off-axis angle increases, resulting in a change in the crack surface area. The larger the section, the higher the required off-axis compressive load, and the change in section leads to a corresponding change in the off-axis compressive strength of the composite. In order to more visually analyze the accuracy of different criteria in predicting the off-axis compressive strength of composites, a comparison of the average prediction errors of different criteria in predicting the off-axis compressive strength is given in Table 3. As can be seen from Table 3, the Puck criterion has the smallest prediction error for these two composite unidirectional plates, and the prediction errors of the improved Chang–Chang criterion for the off-axis compressive strengths of T800H/Epoxy#2500 and IM7/8552 are 12.14% and 15.01%, respectively, which are within the engineering acceptable error range, and are not much different from that of the original 2D Chang–Chang criterion, but the prediction for T800H/Epoxy#2500 is better than the original criterion. The improved Chang–Chang criterion performs poorly in predicting the out-of-plane compressive strength of IM7/8552 composite materials, with an error reaching 15.01%. This error level is not unusual compared to the general performance of existing classical failure criteria—for example, the widely used Hashin criterion can exhibit errors of 20–30% in predicting out-of-plane compression for some composite systems [22,46,47]. This indicates that most current failure models still have inherent limitations in accurately capturing the out-of-plane compression behavior of high-performance carbon fiber composites. The primary causes of prediction deviations can be summarized as follows: First, existing failure theories inadequately characterize failure mechanisms under complex stress states, particularly failing to fully account for the progressive modulating effect of coupled damage. As noted by Gu [44], matrix damage and fiber–matrix interface debonding during off-axis loading induce stress redistribution and trigger multistage failure behavior, while the three-dimensional Chang–Chang criterion improved in this study still employs a one-time failure criterion, failing to effectively reflect coupled effects such as the weakening of transverse load-bearing capacity $\sigma_{22}$ by shear damage $\tau_{12}$ during damage evolution. Second, historical experimental data themselves may contain unidentified systematic errors during testing or recording. Although difficult to trace and verify due to limitations in original conditions, this may also be a source of some deviations.

### 5.2. The Effect of Mechanical Parameters on the Failure Envelope Curve

#### 5.2.1. Effect of Mechanical Parameters on Failure Envelopes at σ_11_-σ_22_ Stress States

**Figure 6:**
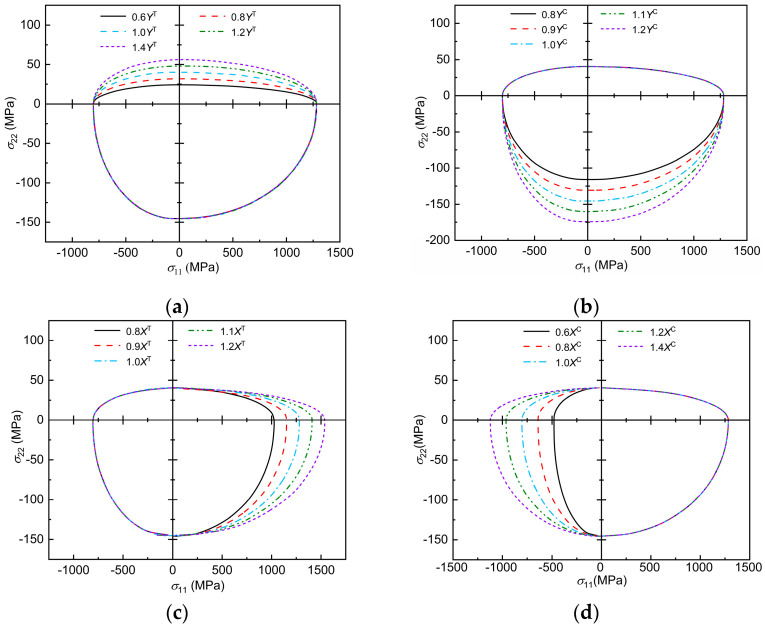
Effect of mechanical parameters on the failure envelope curve: (**a**) trend of envelope curve with Y^T^; (**b**) trend of envelope curve with Y^C^; (**c**) trend of envelope curve with X^T^; (**d**) trend of envelope curve with X^C^.

The envelope curves of transverse and longitudinal damage interactions, predicted by the 3D Chang–Chang criterion, considering transverse and longitudinal interactions under the influence of material mechanical parameters, are analyzed using E-Glass/MY750 as an example, as shown in Figure 6. Note that the highest point of the envelope curve is the transverse tensile basic strength of the material, the lowest point is the transverse compressive basic strength, and the leftmost and rightmost points are the longitudinal compressive and tensile basic strengths, respectively. The effect of the transverse tensile basic strength on the transverse and longitudinal damage envelope curves predicted by the three-dimensional Chang–Chang criterion considering the transverse and longitudinal interactions can be seen in Figure 6a, where the damage envelope becomes more diffuse with increasing $Y_{t}$ in the $\sigma_{11}>0$ region and in the $\sigma_{11}<0$ region, where $Y_{t}$ has no effect on the damage envelope. From Figure 6b, it can be seen that, as $Y_{c}$ increases, the damage envelope becomes more diffuse at $\sigma_{11}<0$ and only affects the damage envelope in this region. From the Figure 6c, it can be seen that, as $X_{t}$ increases, the damage envelope becomes more and more dispersed in the region $\sigma_{22}>0$ and only affects the damage envelope in this region. From the Figure 6d, it can be seen that the damage envelope becomes more and more dispersed in the region of $\sigma_{22}<0$ as $X_{c}$ increases and only affects the damage envelope within this region.

#### 5.2.2. Effect of Mechanical Parameters on Failure Envelopes at σ_11_-τ_12_ Stress States

**Figure 7:**
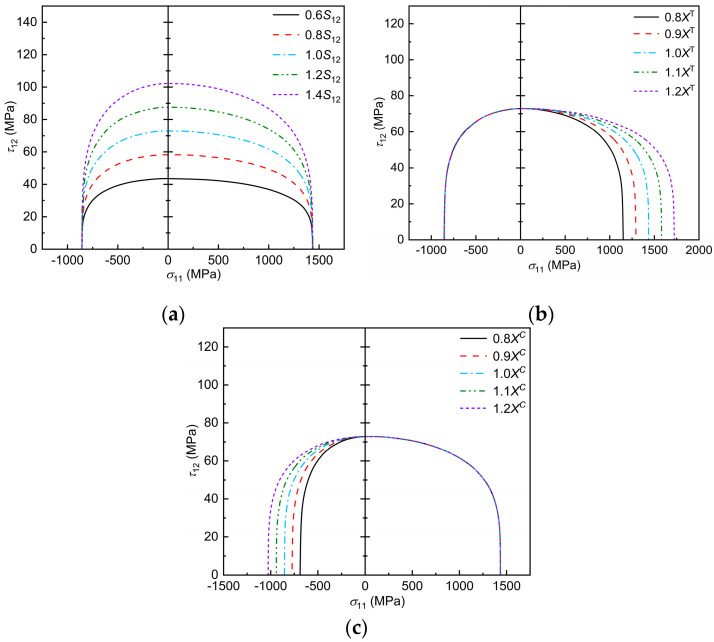
Effect of mechanical parameters on the failure envelope curve: (**a**) trend of envelope curve with *S*_12_; (**b**) trend of envelope curve with *X*^T^; (**c**) trend of envelope curve with *X*^C^.

T300/BSL914C is used as an example to analyze the Chang–Chang criterion envelope curve after improvement under the influence of mechanical parameters, as shown in Figure 7. Note that the highest point of the envelope curve corresponds to the in-plane shear strength of the material, and the left and right intersections with the *x*-axis are the fundamental longitudinal compressive and tensile moduli of the material. The effect of the in-plane shear fundamental strength on the longitudinal damage envelope curves, predicted by the three-dimensional Chang–Chang criterion, considering transverse–longitudinal interactions, can be seen in Figure 7a, where the damage envelope becomes more divergent with increasing $S_{12}$ in the region of $\sigma_{11}>0$. As can be seen in Figure 7b, as $X_{t}$ increases, the damage envelope becomes more diffuse in the $\sigma_{11}>0$, $\tau_{12}>0$ region, while it remains unchanged in the $\sigma_{11}>0$, $\tau_{12}<0$ region. It can be seen from the Figure 7c that, as $X_{c}$ increases, the damage envelope becomes more and more divergent in the region of $\sigma_{11}>0$, $\tau_{12}<0$, while it remains unchanged in the region of $\sigma_{11}>0$, $\tau_{12}>0$.

#### 5.2.3. Effect of Mechanical Parameters on Failure Envelopes at σ_22_-σ_33_ Stress States

**Figure 8:**
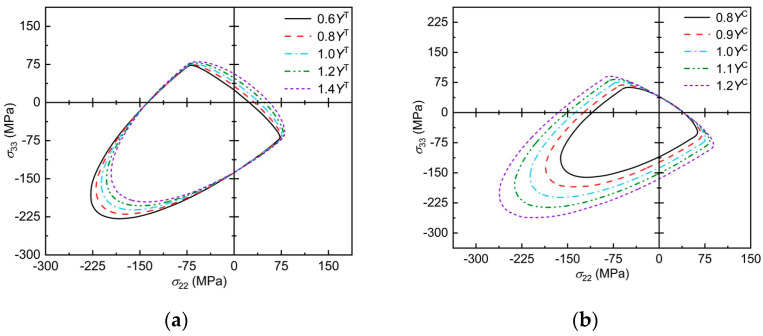
Effect of mechanical parameters on the failure envelope curve: (**a**) trend of envelope curve with *Y*^T^; (**b**) trend of envelope curve with *Y*^C^.

Taking E-glass/LY556 as an example, the improved Chang–Chang criterion envelope curve under the influence of mechanical parameters is analyzed as shown in Figure 8. Note that the right intersection of the envelope curve with the *x*-axis and the upper intersection of the *y*-axis corresponds to the basic longitudinal tensile strength of the material, and the left intersection with the *x*-axis and the lower intersection of the *y*-axis corresponds to the basic longitudinal compressive strength of the material. Figure 8a shows that, with increasing transverse tensile strength, the failure envelope curves become more and more dispersed along the upper right and lower left, and the intersection points with the negative half-axis of the transverse axis and the negative half-axis of the longitudinal axis remain unchanged. Figure 8b shows that, with the increasing of transverse compressive strength, the failure envelope curve does not diverge along the upper right direction but spreads outward in other directions.

#### 5.2.4. Effect of Mechanical Parameters on the Failure Envelope at σ_11_-σ_22_ = σ_33_ Stress States

**Figure 9:**
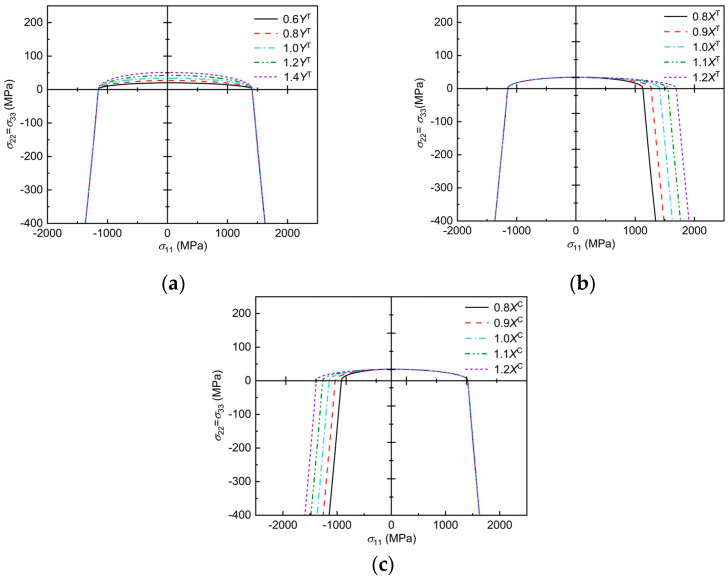
Effect of mechanical parameters on the failure envelope: (**a**) trend of envelope curve with Y^T^; (**b**) trend of envelope curve with X^T^; (**c**) trend of envelope curve with X^C^.

Taking S-glass/epoxy as an example, the Chang–Chang criterion envelope curves after improvement under the influence of mechanical parameters are analyzed as shown in Figure 9. Note that the right intersection of the envelope curve with the *x*-axis corresponds to the basic longitudinal tensile strength of the material, the upper intersection with the *y*-axis corresponds to the basic transverse tensile strength of the material, and the left intersection with the *x*-axis corresponds to the basic longitudinal compressive strength of the material. As shown in Figure 9a, with the increasing transverse tensile strength $Y_{t}$, the failure envelope curve spreads upward, which is manifested by the increasing value of the intersection point with the vertical axis. As shown in Figure 9b, with the increasing longitudinal tensile strength $X_{t}$, the failure envelope curve spreads to the right, which is manifested by the increasing value of the intersection point with the positive semi-axis of the transverse axis. As shown in Figure 9c, with the increasing longitudinal compressive strength $X_{c}$, the failure envelope curve spreads to the left, which is manifested by the increasing absolute value of the corresponding value at the intersection with the negative half-axis of the transverse axis.

#### 5.2.5. Effect of Mechanical Parameters on the Failure Envelope at the σ_11_ = σ_22_ = σ_33_-τ_12_ Stress State

**Figure 10:**
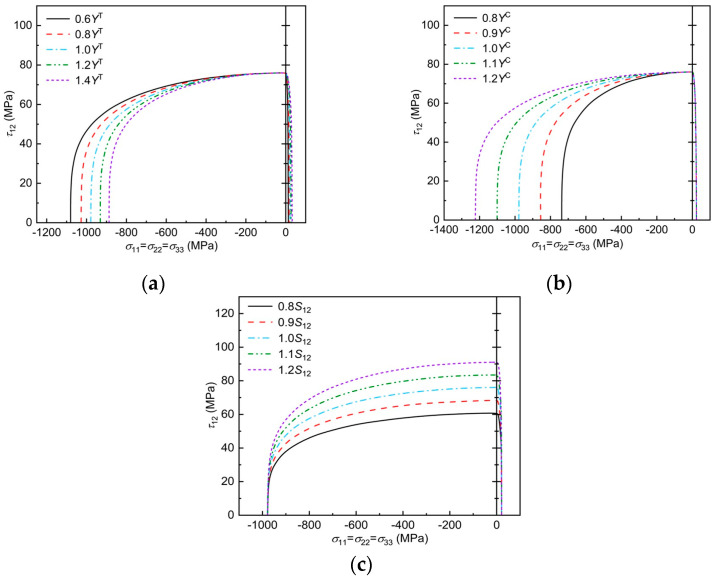
Effect of mechanical parameters on the failure envelope curve: (**a**) trend of envelope curve with Y^T^; (**b**) trend of envelope curve with Y^C^; (**c**) trend of envelope curve with S_12_.

T300/PR319 is used as an example to analyze the Chang–Chang criterion envelope curve after improvement under the influence of mechanical parameters, as shown in Figure 10. Note that the intersection of the envelope curve with the *y*-axis corresponds to the transverse in-plane shear strength of the material, the value at the intersection with the positive half-axis of the *x*-axis corresponds to the transverse tensile strength of the material, and the value at the intersection with the negative half-axis of the *x*-axis corresponds to the transverse compressive strength of the material. As shown in Figure 10a, with the increasing transverse tensile strength $Y_{t}$, the failure envelope curve spreads simultaneously along the left and right directions of the transverse axis, which is manifested by the increasing value of the intersection with the right side of the transverse axis and the decreasing value of the intersection with the left side of the transverse axis. As shown in Figure 10b, with the increasing transverse compression strength $Y_{c}$, the failure envelope curve spreads to the left, which is manifested by the decreasing value of the intersection point with the negative half-axis of the transverse axis. As shown in Figure 10c, as the in-plane shear strength $S_{12}$ continues to increase, the failure envelope curve spreads upward, as evidenced by the increasing value of the intersection with the upper half-axis of the vertical axis.

### 5.3. The Effect of Mechanical Parameters on the Prediction of Off-Axis Tensile Strength

**Figure 11:**
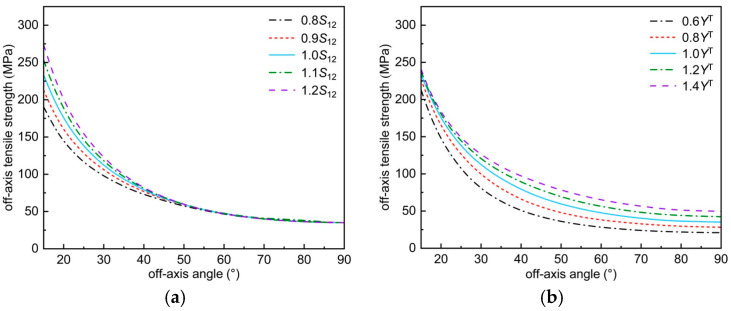
Effect of mechanical parameters on the envelope curve of off-axis tensile failure: (**a**) trend of envelope curve with S_12_; (**b**) trend of envelope curve with Y^T^.

The effect of material parameters on the off-axis tensile strength predicted by the modified Chang–Chang criterion is analyzed using T800H/Epoxy#2500 as an example, as shown in Figure 11. It is worth noting that the off-axis strength is the transverse tensile strength when the off-axis angle is 90°. And other mechanical parameters have no effect on the prediction of off-axis tensile strength.

Figure 11a shows the effect of in-plane shear strength on the off-axis tensile strength predicted by this criterion. It can be seen from Figure 11a that the off-axis tensile strength predicted by this criterion increases with increasing $S_{12}$. From Equation (45) of the proposed criterion, it can be seen that the inclusion term is independent of the other terms, and the predicted value of the off-axis tensile strength increases with the increase of $S_{12}$. From the point of view of material properties, although the increase cannot change the inherent brittleness of the material, it can impede the shear slip between the fibers to a certain extent, thus increasing the off-axis tensile strength.

Figure 11b shows the effect of transverse tensile strength on the off-axis tensile strength predicted by this criterion. From Figure 11b, it can be seen that the predicted off-axis tensile strength increases with increasing $Y_{t}$. This phenomenon is evident from the point of view of material strength theory. From the point of view of material properties, as the inherent brittleness of the material decreases with increasing constants, then the stress concentration caused by small internal cracks decreases, resulting in an increase in the off-axis tensile strength.

### 5.4. The Effect of Mechanical Parameters on the Prediction of Off-Axis Compression Strength

**Figure 12:**
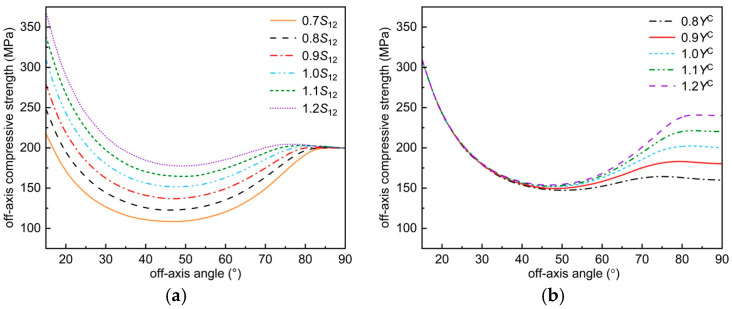
Effect of mechanical parameters on the envelope curve of off-axis compression failure: (**a**) variation trend of envelope curve with *S*_12_; (**b**) variation trend of envelope curve with *Y*^C^.

The effect of material parameters on the off-axis compressive strength predicted by the improved Chang–Chang criterion is analyzed using AS4/3501-6 as an example, as shown in Figure 12. It is worth noting that, when the off-axis angle is 90°, the off-axis compressive strength is the transverse compressive strength. And other mechanical parameters have no effect on the off-axis compression strength prediction.

Figure 12a shows the effect of in-plane shear strength on the off-axis compressive strength predicted by this criterion. It can be seen from Figure 12a that the off-axis compressive strength predicted by this criterion increases with the increase of $S_{12}$. From the strength theory, the off-axis compressive damage of composites is actually caused by shear, and the increase of shear strength certainly increases the off-axis compressive strength of composites.

Figure 12b shows the effect of transverse compressive strength on the off-axis compressive strength predicted by this criterion. It can be seen from Figure 12b that the off-axis compressive strength predicted by this criterion increases with the increase of $Y_{c}$. This phenomenon is evident from the point of view of material strength theory; from the point of view of material properties, as the brittleness increases, the inherent brittleness of the material increases as well, thus increasing the off-axis compressive strength of the composite.

## 6. Conclusions and Future Work Prospects

In this work, based on Chang–Chang criterion, a generalized Mohr failure envelope function is used to obtain the three-dimensional failure function considering the coupling of transverse and longitudinal failure modes by expanding the $\left(\sigma_{ii},\tau_{ij}\right)$ stress on the failure surface as an independent variable in the form of a power series and determining the unknown coefficients according to the damage conditions. Finally, the following important conclusions are found:

1. By incorporating the coupling effects between transverse and longitudinal failure modes, the improved Chang–Chang criterion significantly enhances the accuracy of failure prediction for composite materials under complex stress conditions, such as transverse compression, longitudinal tension, and triaxial compression in three-dimensional stress states. Compared to traditional Chang–Chang, Hashin, and Puck criteria, the proposed criterion demonstrates superior performance. Its applicability is particularly evident in multiaxial stress environments, exhibiting strong potential for application in aerospace composite structures such as wing root attachments and spar joints, where accurate failure prediction under multidimensional loading is critical.
2. By considering the coupling between transverse and longitudinal failure mechanisms, the improved guideline significantly enhances the predictive accuracy of composite materials’ out-of-plane tensile strength, particularly in accurately capturing the transition behavior of failure modes within small out-of-plane angles. Compared to other established standards, it more effectively captures mode-of-failure transitions and demonstrates better consistency with experimental data. These improvements offer practical value for the design and evaluation of automotive composite components, such as chassis elements and battery enclosures, which frequently encounter out-of-plane loading conditions.
3. The improved criterion improves the rationality of failure prediction under biaxial stress conditions by introducing the problem that the traditional criterion of finite biaxial transverse compressive strength ($Y_{\mathrm{cbi}}$) does not close the failure envelope under biaxial compressive stress, which provides a more accurate failure assessment tool for the safe design of composites in complex engineering environments. For example, in wind turbine blade root connections, accurate failure prediction under biaxial compression is critical for structural integrity and safety.

The current framework could be extended to explore more complex scenarios, such as the influence of mild anisotropy, dynamic loading effects, and progressive damage interactions. A refined understanding of these aspects may further improve the model’s predictive accuracy and applicability.

## Figures and Tables

**Table 1:** Elastic constants and strength values of composites.

Material	Modulus/GPa		Poisson’s Ratio	Strength/MPa				
	*E*_1_	*E*_1f_	*v*_12_	*X*_t_	*X*_c_	*Y*_t_	*Y*_c_	*S*_12_
E-glass/MY750 [37]	45.6	74	0.278	1280	800	40	145	73
T300/BSL914C [28]	138	230	0.28	1433.6	853	27	200	73
E-glass/LY556 [28]	53.48	80	0.291	1140	570	40	137.4	61.2
S-glass/epoxy [38]	52	87	0.3	1410	1147	63	180	72
A-S carbon/epoxy [38]	140	231	0.3	2000	1500	38	150	72
T300/PR319 [38]	129	231	0.318	1378	950	40	125	60
Flax-polyester [39]	15.3	4.1	0.269	143	-	13.3	-	20
T800H/Epoxy#2500 [40]	-	-	-	1319.5	1045	51.7	244	95.1
IM7/8552 [41]	171	9.1	0.32	2323	1017	160	255	89.6
AS4/3501-6 [25]	126	11	0.49	2300	1725	60.2	285	73.4

**Table 2:** Comparison of mean errors in off-axis tensile strength predicted by different failure criteria.

Material	Chang–Chang (Coupled 3D)	Chang–Chang 2D and 3D	Hashin	Puck
Flax-ployester	4.71%	6.07%	2.36%	2.54%
T800H/Epoxy#2500	7.36%	8.84%	7.16%	6.16%

**Table 3:** Comparison of mean errors in off-axis compressive strength predicted by different failure criteria.

Material	Chang–Chang (Coupled 3D)	Chang–Chang 2D and 3D	Hashin	Puck
T800H/Epoxy#2500	12.14%	13.90%	15.28%	10.36%
IM7/8552	15.01%	14.75%	13.05%	3.48%

## Data Availability

The original contributions presented in the study are included in the article, further inquiries can be directed to the corresponding author.
